# Comparison of four skin preparation strategies to prevent catheter-related infection in intensive care unit (CLEAN trial): a study protocol for a randomized controlled trial

**DOI:** 10.1186/1745-6215-14-114

**Published:** 2013-04-27

**Authors:** Véronique Goudet, Jean-François Timsit, Jean-Christophe Lucet, Alain Lepape, Dorothée Balayn, Sabrina Seguin, Olivier Mimoz

**Affiliations:** 1 Medical Intensive Care Unit, CHU de Poitiers, Poitiers, France; 2 Inserm U823 “Outcome of cancers and critical illness”, Albert Bonniot Institute, La Tronche CEDEX, 38076, France; 3 Université Grenoble 1, Medical ICU, Albert Michallon Hospital, Grenoble, France; 4 Infection Control Unit, Bichat - Claude Bernard University Hospital, Assistance-Publique Hôpitaux de Paris and Université Paris Diderot, Sorbonne Paris Cité, France; 5 Université Claude-Bernard Lyon 1, Medical-surgical ICU, Centre hospitalier Lyon sud, Lyon, France; 6 CHU de Poitiers, Université de Poitiers and INSERM U1070 “Pharmacology of Anti-Infective Drugs”, Poitiers, France; 7 Service d’Anesthésie Réanimation, CHU de Poitiers, Poitiers, 86000, France

**Keywords:** Central venous catheter, Arterial catheter, Colonization, Bacteremia, Severely ill patient

## Abstract

**Background:**

Catheter-related infection is the third cause of infections in intensive care units (ICU), increasing the length of stay in ICU and hospital, mortality, and costs. Skin antisepsis is one of the most prevalent preventive measures. In this respect, it would appear preferable to recommend the use of alcoholic povidone iodine or chlorhexidine rather than aqueous povidone iodine. However, the data comparing chlorhexidine to povidone-iodine, both of them in alcoholic solutions, remain limited. Moreover, the benefits of enhanced cleaning prior to disinfection of skin that is not visibly soiled have yet to be confirmed in a randomized study.

**Methods:**

A prospective multicenter, 2×2 factorial, randomized-controlled, assessor-blind trial will be conducted in 11 intensive care units in six French hospitals. All adult patients aged over 18 years requiring the insertion of at least one peripheral arterial catheter and/or a non-tunneled central venous catheter and/or a hemodialysis catheter and/or an arterial pulmonary catheter will be randomly assigned to have all their catheters cared with one of four skin preparation strategies (2% chlorhexidine/70% isopropyl alcohol or 5% povidone iodine/69% ethanol with or without prior skin scrubbing). At catheter removal, catheter tips will be quantitatively cultured. Sets of aerobic and anaerobic blood cultures will be routinely obtained when a patient has fever, hypothermia, or other indications. In case of suspected catheter-related infection the patient’s form will be reviewed by an independent adjudication committee. We plan to enroll 2,400 patients (4,800 catheters). The main objective is to demonstrate that use of 2% alcoholic chlorhexidine compared to 5% alcoholic povidone iodine in skin preparation lowers the rate of catheter-related infection. The second endpoint is to demonstrate that enhanced skin cleaning prior to disinfection of skin that is not visibly soiled does not reduce catheter colonization. Other outcomes include comparison of skin colonization at catheter insertion site, comparison of catheter colonization and catheter-related bacteremia taking place during implementation of the four strategies of skin preparation, and cutaneous tolerance, length of hospitalization, mortality, and costs.

**Discussion:**

This study will help to update recommendations on the choice of an antiseptic agent to use in skin preparation prior to insertion of a vascular catheter and, by extension, of an epidural catheter and it will likewise help to update recommendations on the usefulness of skin scrubbing prior to disinfection when the skin is not visibly soiled.

**Trial registration:**

Clinicaltrials.gov number NCT01629550

## Background

Catheter-related infections represent the third cause of infections in intensive care units (ICUs) after pneumonia and complicated intra-abdominal infections [1]. These infections increase the length of ICU and hospital stay, mortality, and costs. Additional costs as high as $30,000 per survivor have been reported, including 1 extra week in the ICU and 2 to 3 additional weeks in the hospital [2]. Attributable mortality rates range from 0 to 35%, depending on the degree of control for severity of illness [2].

The physiopathology of catheter infection is now more clearly understood [2]. For short-term catheters such as those inserted in ICUs, catheter tip colonization arises during catheter insertion or, less frequently, by migration of skin organisms from the insertion site into the cutaneous catheter tract during catheter maintenance. The density of micro-organisms at catheter insertion site is therefore a major risk factor for short-term catheter-related infection, while skin antisepsis is one of the most effective preventive measures.

Povidone iodine and chlorhexidine are the two most commonly used antiseptic agents, both of them being available as aqueous and alcoholic formulations. Their respective efficacy in preventing catheter colonization and bloodstream infections has been compared in numerous studies. One meta-analysis including eight randomized trials and 4,143 short-term catheters, published in 2002, found that chlorhexidine solutions either in aqueous or alcoholic formulations significantly reduced catheter-related bloodstream infections by approximately 50% (RR, 0.51 (95% CI, 0.27-0.97)) compared to 10% for aqueous povidone iodine [3]. These findings were subsequently confirmed in other trials [4-6], leading in many countries, the USA [7], England [8], and France [9], to cessation of the recommendation of aqueous povidone iodine in catheter care. The superiority of chlorhexidine has been linked by some authors to a synergistic effect with alcohol, which has also been demonstrated with povidone iodine. Compared to aqueous 10% povidone-iodine, a double application of 5% povidone iodine in 69% ethanol reduced the incidence of catheter colonization (RR, 0.38 (95% CI, 0.22-0.65)) and catheter-related infection (RR, 0.34 (95% CI, 0.13-0.91)) in a randomized unit-crossover study that included 223 central venous catheters [10]. No significant effect was observed on bloodstream infections, but the study was underpowered to explore this issue.

Two French trials have directly compared alcoholic formulations of chlorhexidine and povidone iodine. The first one included 481 evaluable central venous catheters inserted into jugular or subclavian veins [11]. Use of a combination of 0.25% chlorhexidine gluconate, 0.025% benzalkonium chloride, and 4% benzylic alcohol was associated with a reduction by half in the incidence of catheter colonization (11.6% *vs.* 22.2% *P*= 0.002; incidence density, 9.7 *vs*. 18.3 per 1,000 catheter-days). A trend toward lower rates of catheter-related bloodstream infection was likewise noted (1.7% *vs.* 4.2% *P*= 0.09; incidence density, 1.4 *vs.* 3.4 per 1,000 catheter-days), notwithstanding the fact that the study was not powered to adequately explore this issue. However, lack of skin scrubbing (which is strongly recommended in France) prior to antiseptic administration, completion of the study in a single unit, and use of a combination of three compounds in the chlorhexidine arm all constituted obstacles to generalization of these findings. The second trial was a single-center before-after study comparing the efficacy of the same antiseptics as in the previous trial and included 435 central venous catheters in the povidone iodine arm and 371 central venous catheters in the chlorhexidine arm [12]. The switch from alcoholic povidone iodine to the chlorhexidine-based antiseptic solution was associated with a reduction in catheter colonization (11.2 *vs.* 15.5 per 1,000 catheter days, *P*=0.041) and a pronounced decline in catheter-related bacteremia (1.4 *vs.* 3.0 per 1,000 catheter days, *P*=0.052). Moreover, use of alcoholic povidone iodine was independently associated with an increased risk of catheter colonization or infection in multivariate cox model analysis (1.48, 95% CI, 1.01-2.15, *P*=0.043). This study nonetheless has several limitations, including its design, but it succeeds in confirming the superiority of chlorhexidine-based solutions over povidone iodine, even in alcoholic formulations, in care of central venous catheters.

The superiority of chlorhexidine over povidone iodine has been linked to quick bactericidal activity, poor inactivation by blood and other protein-rich biomaterials present on skin, and long-term antimicrobial suppressive activity. These findings have led the Centers for Disease Control and Prevention (CDC) to include in their 2011 recommendations [7] the use of alcoholic chlorhexidine at a concentration >0.5% as a first-line antiseptic in catheter care. However, they pointed out that data comparing chlorhexidine and povidone-iodine in alcoholic solutions are limited and recommended the organization of a large-scale study comparing alcoholic formulations of chlorhexidine and povidone iodine.

In addition, skin scrubbing reduces bacterial load and the amount of protein-rich biomaterials present on skin and may thereby enhance the efficacy of antiseptics. However, the benefits of enhanced skin cleaning prior to disinfection of skin that is not visibly soiled have never been confirmed in a randomized study. Its use, recommended in only a few countries such as France, is based on the results of a study showing a link between the amount of skin colonization before antiseptic application and catheter colonization [13]. These findings offer only indirect evidence in favor of skin scrubbing before skin antisepsis. The French Society of Hospital Hygiene (SF2H) recommends the systematic skin scrubbing prior to antisepsis while the CDC, on the other hand, indicates that antiseptics could be directly applied on clean skin [7].

The objectives of this study are to demonstrate in adult ICU patients (1) the ability of 2% chlorhexidine/70% isopropyl alcohol to decrease the rate of catheter-related infection compared with the use of 5% povidone iodine/69% ethanol and (2) the inability of skin scrubbing prior to disinfection to reduce catheter colonization when the skin is not visibly soiled.

## Methods

### Ethics statement

The study was approved by the local ethics committee (CPP Ouest III, France) on 21 May 2012 (Protocol# 12.02.06). Each patient will have oral and written information concerning the study design and outcomes. If the information cannot be delivered to the patient, it will be delivered to his/her relatives.

### Study design

The CLEAN study is a prospective multicenter, 2×2 factorial, randomized-controlled, assessor-blind trial involving 11 intensive care units in five French University-affiliated hospitals and one French general hospital and is designed to compare two antiseptics (2% chlorhexidine/70% isopropyl alcohol and 5% povidone iodine/69% ethanol) with or without prior skin scrubbing. Due to the different colors of the two solutions, nurses and physicians will not be blinded to the antiseptic agent used. However, the microbiologists assigned to process all of the cultures and the adjudication committee tasked with reviewing the outcomes other than colonization will be unaware of the patients’ arms.

Inclusions opened at the end of October 2012 and will close approximately 14 months later.

### Patients

All consecutive adult patients aged over 18 years, hospitalized in one of participating ICUs for an expected length of stay of >2 days and requiring insertion of at least one peripheral arterial catheter and/or a non-tunneled central venous catheter and/or a hemodialysis catheter and/or an arterial pulmonary catheter will be included in the trial. Exclusion criteria will be catheters inserted outside the ICUs, patients with a history of allergy to any of the antiseptic agents studied, patients likely to die within 48 h after admission, and use of catheters coated with antimicrobial agents.

### Randomization

Patients will be randomly assigned in a 1:1:1:1 ratio to have all their catheters cared with either 2% chlorhexidine/70% isopropyl alcohol (Chloraprep™, CareFusion™, Voisins le Bretonneux, France) or 5% povidone iodine/69% ethanol (alcohol Bétadine™, MEDA™ Pharma SAS, Paris, France) with or without prior scrubbing with an antiseptic scrub. Randomization will be stratified by hospital with the use of a web-based random-number generator producing permuted blocks to address for potential interhospital heterogeneity.

### Skin antisepsis

Antiseptic agents used will be:

– 5% povidone iodine/69% ethanol preceded or not by scrubbing insertion site with a detergent (4% povidone iodine, Betadine™ Scrub, MEDA™ Pharma).

– 2% chlorhexidine/70% isopropyl alcohol (applicator sponge soaked with the antiseptic, Chloraprep™) preceded or not by scrubbing insertion site with a detergent (4% chlorhexidine, Hibiscrub™, Molnlycke Health Care, Wasquehal, France).

According to the randomization arm, antiseptic strategy during catheter insertion and maintenance will be:

– One-step procedure. The physician who will insert the catheter will disinfect the skin using maximal barrier precautions (surgical hand antisepsis and wearing a gown, cap, mask, and sterile gloves). Antiseptic will be applied moving back and forth (2% chlorhexidine/70% isopropyl alcohol) or by circular movements (5% povidone iodine/69% ethanol) for 30 s, starting at the catheter insertion site and then expanding to the entire work area. Large sterile drapes will be applied once the work area dries. Catheters will then be inserted without any further application of antiseptic.

– Four-step procedure. The work area will be scrubbed by a nurse using sterile gauze soaked with detergent applied by circular movements for 15 s, rinsed with sterile water, and dried with sterile gauze. Antiseptic and large sterile drapes will then be applied by the physician using maximal barrier precautions as described in the one-step procedure. Finally, catheters will be inserted without any further application of antiseptic.

In order to avoid any risk of confusion, antiseptic kits containing all the products needed according to randomization arm will be available in the patient’s room; similarly, a poster showing how to carry out skin preparation will be displayed in the patient's room. Training for healthcare providers designed to homogenize skin preparation practices between units will be held before starting inclusions. A clinical research associate will be available at each participating Hospital to help with data collection and to monitor for adequate practices.

### Catheter insertion and maintenance

All study centers are to follow French recommendations (similar to those of the CDC) for catheter insertion and care. Use of the subclavian vein (except for hemodialysis catheter) and radial artery will be encouraged whenever possible. Catheters will be placed percutaneously using the Seldinger technique. Ultrasound guidance shall be used at the discretion of the attending physician. Before catheter insertion, the skin will be disinfected with the randomized antiseptic procedure assignment. After insertion, catheters will be dressed with semi-permeable non-chlorhexidine transparent dressing. For each unit, the same catheter and dressing types will be used for the duration of the study period. Catheter insertion sites will be inspected every day for signs of infection. Dressing will be changed every 72 h to 7 days according to center protocol or earlier if leaking, soiled, or wet (except for the first dressing changed 24 h after catheter insertion). Manipulation of lines and three-way stopcocks will be carried out with gauze moistened with 5% povidone iodine/69% ethanol in povidone iodine arms and 0.5% chlorhexidine/67% alcohol (Hibitane Champ™, Molnlycke Health Care, Wasquehal, France) in the chlorhexidine arm.

Use of antiseptic-containing dressings, topical antimicrobial ointments, antimicrobial filters, and line locks will not be allowed. Blood sampling through the central venous line will not be permitted. The decision to remove the catheter will be made solely by the patient’s physician, but ablation of the catheter when it is losing its usefulness will be encouraged. Patients leaving the ICU with their central venous catheter will undergo one peripheral and one central line blood culture and will be followed for 3 days after ICU discharge to monitor possible infectious complications. Any catheter infection occurring >3 days after ICU discharge will not be taken into account due to the risk of protocol violation during catheter manipulation or of maintenance by untrained healthcare providers.

### Cultures

Catheters will be removed aseptically and their distal 5cm will be sectioned with sterile scissors and placed in a sterile tube before being carried to the microbiology laboratory. Catheters will be cultured quantitatively by using a simplified technique of quantitative broth dilution culture [1,14].

Sets of aerobic and anaerobic blood cultures will be routinely obtained when a patient has fever, hypothermia, or other indications such as chills or sudden shock.

Skin colonization will be evaluated before catheter removal by using semi-quantitative insertion-site cultures. The insertion site will be sampled by pressing on the skin for 10 s a sterilized nutritive trypticase-soy agar plate containing antiseptic neutralizing agents (Count-tact™, 3P Pack+, Biomerieux™, Crapone, France).The plate will then be sent to the local microbiology laboratory and cultured for 48 h. The number of colony-forming units will be counted and classified as: sterile; 1 to 9 CFU; 10 to 49 CFU, 50 to 99 CFU, or ≥100 CFU per agar plate as previously done [9,15].

All cultures will be processed by the clinical microbiology laboratory according to standard methods without awareness of the randomization arm.

In cases of suspected catheter-related infection (that is, all the catheters removed with systemic inflammatory response syndrome (SIRS) or local signs of infection (pain or purulence) or positive catheter tip culture, or positive blood culture sampled 48 h before until 48 h after the catheter removal), the patient’s form will be reviewed by an independent adjudication committee. In the event of doubt, a complete blind patient record will be prepared by the clinical research associates. Two independent experts blinded to the randomization arm will classify these episodes according to infection definitions (see below). In case of disagreement, the opinion of a third expert will be sought.

### Data collection

– For each center: number of hospital beds, type of ICU (medical, surgical or mixed, number of ICU beds, number of admissions a year in ICU, mean Simplified Acute Physiology Score II (SAPS II) [16], mean length of stay in ICU, and details of procedures for catheter insertion and maintenance.

– For each patient included: demographic data (age, sex, weight, and height), SAPS II and Sequential Organ Failure Assessment (SOFA) [1] scores at admission, length of mechanical ventilation, use of catecholamines, clinical and microbiological data needed to confirm the diagnostic of catheter-related infection.

– For each catheter: catheter insertion date, insertion site, catheter removal date, use of ultrasound guidance, insertion using guidewire exchange, experience of the operator, IGS II and SOFA scores at catheter insertion, antibiotics at catheter insertion, antibiotics used during catheter stay, administration of parenteral nutrition, propofol or blood product administration, reason(s) for catheter withdrawal, catheter tip culture result, blood culture result, local signs at catheter insertion site, status of dressing at catheter removal, evolution of signs of infection at catheter withdrawal, number and results of blood cultures drawn within 48 h around catheter removal during catheter stay, and skin colonization at removal of catheter assessed using semi-quantitative insertion site culture.

Three audits will be conducted in each center, each of them including four catheter insertions and 10 dressing changes, the first one during the first 2 months of study, the second one after inclusion of 100 patients, and the last one after inclusion of 200 patients. The costs of each antiseptic strategy will be evaluated based on material and time needed for skin preparation.

### Definitions

French [9] and American [7] guidelines will be used by the blind adjudication committee.

– Catheter colonization will be defined as a quantitative culture tip showing at least one micro-organism at a concentration of 1,000 colonies forming 1 unit per milliliter (cfu/mL) or more.

– Catheter-related sepsis without bacteremia will be defined according to the combination of (1) fever (body temperature ≥38.5°C) or hypothermia body temperature ≤36.5°C), (2) catheter colonization, (3) regression of fever or hypothermia within the 48 h following catheter removal and without any change of antimicrobial therapy, or presence of pus at catheter insertion site, and (4) no other source of infection identified.

– Catheter-related bloodstream infection will be defined as the combination of (1) fever (body temperature ≥38.5°C) or hypothermia body temperature ≤36.5°C), (2) one or more positive peripheral blood cultures drawn 48 h before or after catheter withdrawal, (3) isolation of the same organism (same species and same susceptibility pattern) from the colonized catheter, or from the catheter insertion site, or a blood-culture differential time-to-positivity of 2 h or more, and (4) no apparent source of bacteremia other than the catheter. In patients with positive coagulase negative staphylococci bacteremia, at least two positive cultures obtained from separate blood samples will be mandatory.

– Major catheter-related infections will be either catheter-related sepsis without bacteremia or catheter-related bloodstream infections.

– Non-cultured catheters or catheters growing under 1,000 cfu/mL will be classified as catheter-related sepsis or bloodstream infections in case of sepsis without or with bacteremia and no other detectable source.

– Non-cultured catheters will be classified as not colonized unless there was sepsis with no other detectable cause.

### Outcomes

The main endpoint will be to demonstrate that compared to 5% povidone iodine/69% ethanol, use of 2% chlorhexidine/70% isopropyl alcohol for skin preparation lowers the rate of major catheter-related infection. The second endpoint will be to demonstrate that skin scrubbing prior to antiseptic application does not reduce catheter colonization when the skin is not visibly soiled. Other outcomes include comparison of colonization at catheter insertion site, catheter colonization, and catheter-related bacteremia, as well as cutaneous tolerance, length of hospitalization, mortality, and costs between the four strategies of preparation of skin. In particular, a comparison between 2% chlorhexidine/70% isopropyl alcohol not preceded by scrubbing and 5% povidone iodine/69% ethanol preceded by scrubbing will be carried out.

### Assessment endpoints

Density incidence of major catheter-related infection, catheter colonization, catheter-related bloodstream infection, and skin colonization at catheter insertion site for all catheters and for arterial or central venous catheters only; mortality rate in ICU and in the hospital; length of stay in ICU and in hospital; incidence of cutaneous allergy using the International Contact Dermatitis Research Group scale [17]; cost of each strategy of skin preparation.

### Sample size calculation

Assuming 5% of catheter-related infection rate in the 5% povidone iodine/69% ethanol group (that is, incidence density of 4 per 1,000 catheters days) and the use of at least two catheters per patient on average, 453 patients in each treatment arm will be required to demonstrate a 50% reduction of major catheter-related infection with the use of 2% chlorhexidine/70% isopropyl alcohol, with a power of 80% and a two-sided alpha risk of 5%. We plan to enroll 2,400 patients (4,800 catheters) to take into account the interaction between antiseptic efficacy and skin scrubbing and a maximum patient loss of 6%.

### Statistical analysis

The data will be analyzed blindly with regard to treatment arms. An intention-to-treat analysis is scheduled, and it will include all patients except those with no catheter inserted and those who will have withdrawn their consent to participate in the study. No interim analyses are planned. Demographic data will be described in number and percentage or median and interquartile range for quantitative and qualitative variables and compared with χ^2^ test or Mann–Whitney, as appropriate. Kaplan-Meir curves of the risks of major-CRI and CR-BSI and catheter colonization will be plotted for each treatment group. Proportional hazard assumption will be assessed by plotting Schoenfield’s residuals with time.

Analysis of antiseptic efficacy will be carried out using a marginal Cox model stratified by centers [18]. This model takes into account the censored nature of the data and also accounts for intra-cluster (intra-patient) dependence (more than one catheter per patient) using a robust sandwich covariance matrix. The interaction between scrubbing and antiseptic effect will be sought first by forcing the interaction term in the final model. In the absence of interaction, analysis of skin scrubbing and chlorhexidine effects will be performed independently. For secondary endpoints the analyses will not be controlled for multiple testing.

Comparisons of skin cultures between groups will be assessed through multiple factor ANOVA (Center, scrubbing, antiseptic).

Analyses will be done using SAS 9.x (SAS Institute, Cary, NC,USA) and R (R foundation, Vienna, Austria) softwares.

### Length of patient follow-up

Patients will be followed up for the duration of their stay in ICU. Insofar as possible the catheter will be removed before patients’ discharge from ICU. If not, one peripheral and one through-the-line blood culture will be drawn at discharge and a visit at day 3 will take place in order to enquire about the diagnostic of catheter-related infection.

### End of participation in the study

A patient will leave the study if he (she) refuses to participate to it, if an exclusion criterion appears during hospital stay, if a severe adverse event occurs or if there is an allergy to one of the antiseptic agents.

## Discussion

This study will help to update recommendations on how to prepare the skin prior to insertion of a vascular catheter and, by extension, an epidural catheter, of which it is estimated that approximately 30 million are inserted every year in France. Although chlorhexidine is considered the most effective antiseptic in reducing catheter-related infections, its superiority over alcoholic povidone iodine is based on only a few studies, which have not included enough patients to demonstrate any significant effect on infection. A study adequately powered to demonstrate the superiority of chlorhexidine over alcoholic povidone iodine in reducing major catheter-related infections is warranted (7). Skin scrubbing is rapid (<2 min) and inexpensive (<€1), but since it is performed several million times a year by nurses in French hospitals, it leads to substantial costs.

Study limitations. The possible impact on the findings of different types and concentrations of alcohol contained in both antiseptics studied, of different methods of application of antiseptics and of different antiseptics used for manipulating the lines and of three-way taps shall not be assessed. However, antiseptics will be used in their available formulations and in accordance with the manufacturers’ recommendations.

### Trial status

The trial is currently including patients. The inclusion process started on 26 October 2012 and the number of patients included is 691. The estimated length of inclusion time is 15 months.

## Abbreviations

CDC: Centers for Disease Control and Prevention; CFU: Colony forming unit; CI: Confidence interval; ICUs: Intensive care units; RR: Relative risk; SAPS: Simplified acute physiology score; SF2H: French Society of Hospital Hygiene; SIRS: Systemic inflammatory response syndrome; SOFA: Sequential organ failure assessment.

## Competing interests

O Mimoz has received lecture and consultancy fees from CareFusion, 3 M Company, and Ethicon. JF Timsit has received lecture fees from Carefusion, and lecture fees and research grants from Ethicon and 3 M Company. JC Lucet has received lecture fees from CareFusion and 3 M Company. No other potential conflict of interest relevant to this article has been reported.

## Authors’ contributions

VG and OM drafted the manuscript and contributed to the design of the study. JCL and AL contributed to the design of the study and helped to draft the manuscript. JFT contributed to the design of the study, helped out with the statistical analysis, and helped to draft the manuscript. All authors read and approved the final manuscript.

## Participating centers

Medical and Surgical Intensive Care Unit, University Hospital of Angers (Pr Lasocki and Pr Mercat).

Medical and Surgical Intensive Care Unit, University Hospital of Clermont-Ferrand (Pr Constantin and Pr Souweine).

Medical and Surgical Intensive Care Unit, University Hospital of Lyon (Pr Bohé, Dr Lepape,and Dr Tassin).

Medical and Surgical Intensive Care Unit, University Hospital of Paris Bichat (Pr Montravers and Pr Wolff).

Medical and Surgical Intensive Care Unit, University Hospital of Poitiers (Pr Mimoz, Pr Robert,and Dr Goudet).

Polyvalent Intensive Care Unit, General Hospital of Tourcoing (Dr Alfandari).
